# Clinicopathological Profiles of and Patterns of Recurrence in Triple-Negative Breast Cancer Patients at a Cancer Care Center in Southern India

**DOI:** 10.7759/cureus.63886

**Published:** 2024-07-05

**Authors:** Prudhvi Inampudi, Deepak C Yadlapalli, Muralidhar Gullipalli

**Affiliations:** 1 Medical Oncology, Ganni Subbulakshmi Garu Medical College, Rajahmundry, IND

**Keywords:** triple-negative breast cancer, clinicopathological profile, india, recurrence, breast cancer

## Abstract

Background: Triple-negative breast cancer (TNBC) is characterized by the absence of expression of the estrogen receptor and the progesterone receptor by immunohistochemistry and human epidermal growth factor receptor overexpression absence either by immunohistochemistry or absence of amplification by fluorescence in-situ hybridization. TNBCs tend to have rapid growth when compared to other subtypes of breast cancer. TNBC is associated with higher histologic grade and more advanced disease at presentation. TNBC shows aggressive behavior and a high chance of recurrence.

Aim: The aim was to analyze the clinicopathological profiles of and recurrence patterns in TNBC patients at our institute where most patients are from rural areas.

Methods: This retrospective study was done at a tertiary cancer care center in Southern India where most patients come from rural backgrounds. Institutional Ethics Committee approval was obtained before the study. Case files of all breast cancer patients registered and treated at our center from 2014 to 2019 were retrieved from the medical record department and reviewed. Data from patients diagnosed with triple-negative breast cancer were identified and analyzed.

Results: Among the 841 breast cancer patients registered in our study, 150 (17.8%) were diagnosed with TNBC. The median age of diagnosis was 47 years. The majority of the patients, 89 (59.3%) presented with T2 tumors, and lymph node involvement was observed in 88 (58.6%) cases. Patient distribution based on cancer stage revealed that 77 (51.3%) had early-stage breast cancer (EBC), 70 (46.6%) had locally advanced breast cancer (LABC), and only three patients were categorized as having metastatic breast cancer (MBC). Modified radical mastectomy (MRM) was the preferred surgical approach in 144 (96%) cases, while only four patients underwent breast-conserving surgery (BCS). Adjuvant chemotherapy was administered to 119 (79.3%) patients, with 30 (20%) receiving both neoadjuvant and adjuvant chemotherapy (NACT/ACT). Among those who underwent NACT/ACT, a pathological complete response was observed in five (16.6%) patients out of 30 patients. The median duration of follow-up was 32.8 months. Among all patients, 36 (24%) experienced recurrence, with seven (19.4%) having local recurrence, 24 (66.6%) developing distant metastases, two patients experiencing both local and distant recurrence, and three patients developing contralateral breast cancer. Additionally, three patients experienced a second primary cancer. The most common sites of metastases were the lungs (14), followed by the bone (seven), the liver (four), and the brain (four). Recurrence rates were notably high within the first one to three years post-diagnosis. The median disease-free survival (DFS) of TNBC patients was estimated to be 65.6 months with no statistically significant difference (p=0.174) between EBC and LABC patients.

Conclusion: TNBC is known for its heterogeneity. While it is often regarded as being more responsive to chemotherapy compared to other subtypes of breast cancer, TNBCs tend to behave aggressively, basically due to the underlying aggressive tumor biology. Though there are many treatment options for different subtypes of breast cancer, therapeutic modalities are limited for TNBCs. Aggressive tumor biology with limited treatment options denotes a gap in the development of novel strategies to improve outcomes in this subset of breast cancer patients.

## Introduction

Breast cancer is the most common cancer among women worldwide and in India. According to Globocan 2022, it accounts for 26.6% of all cancer cases among women in India [1]. In 2022, there were 2.3 million women diagnosed with breast cancer and 670,000 deaths globally. Breast cancer had the highest proportion in India, with 192,020 new cases, accounting for 13.6 percent of all patients with over 26 percent in women and 98,337 deaths. It is a heterogeneous disease entity encompassing numerous distinctive histological, immunohistochemical, and gene profile-based subtypes. Based on gene expression profiling, breast cancer is divided mainly into four subtypes: luminal A and luminal B, human epidermal growth factor receptor 2 (HER2) expressing, and basal‑like. However, gene expression profiling is the gold standard for identifying the molecular subtypes of breast cancer but is difficult to use in routine clinical practice due to its high cost.

In day-to-day practice, based on the immunohistochemical expression of the estrogen receptor (ER), progesterone receptor (PR), and human epidermal growth factor receptor 2 (HER2), breast cancer is classified into various molecular subtypes. Triple-negative breast cancer (TNBC) is characterized by the absence of expression of the ER and PR by immunohistochemistry (IHC) and human epidermal growth factor receptor overexpression absence either by IHC or absence of amplification by fluorescence in-situ hybridization. The triple-negative clinical phenotype mostly comprises the basal-like molecular subtype, although triple-negative and basal breast cancers are not synonymous, and substantial heterogeneity exists within TNBCs. TNBCs are characterized by having unique clinical, pathological, and molecular behavior. Gene expression analysis has shown that, most commonly, tumor suppressor gene p53 (TP53) and several DNA repair genes, particularly the breast cancer susceptibility genes (BRCA), are mutated or abnormally expressed in TNBC.

The incidence of TNBC in the Western world is around 12-13% [2,3]. In India, studies suggest a higher TNBC incidence of around 27-31% [4-6]. Unlike hormonal-positive and HER2-positive breast cancers, there are not many approved targeted treatments, especially for patients with TNBC. For advanced and metastatic TNBC, immunotherapy combined with chemotherapy is available, particularly for those patients whose tumors express programmed cell death ligand 1 (PD-L1). Compared to hormonal-positive and HER2-positive breast cancers, TNBC occurs more frequently in younger women. TNBCs tend to have rapid growth when compared to other subtypes of breast cancer [7]. It is associated with higher histologic grade and more advanced disease at presentation. TNBC shows aggressive behavior and a high chance of recurrence. The most common histology associated with TNBC is infiltrating ductal carcinoma [8].

TNBC is associated with aggressive behavior and a high chance of recurrence. Numerous studies in Western literature have discussed TNBCs, consistently emphasizing the unfavorable prognosis associated with this particular subtype of breast cancer [9]. At around three years post-diagnosis in TNBCs, there will be a peak in the risk of distant recurrence and death for TNBC, and then the risk of recurrence decreases rapidly. TNBC has higher relapse rates during these first few years compared to ER-positive breast cancers. However, while ER-positive cancers may continue to recur for many years, TNBCs typically do not [10]. TNBC is more likely to recur in locoregional areas and visceral organs, including the liver, lungs, and brain, at the first recurrence [11,12]. On the other hand, recurrence in the bone will be less in TNBCs when compared to those with hormonal-positive breast cancers [11,12]. Short-term prognosis will be poorer (first five to seven years) in patients with TNBCs when compared with patients with other subtypes of breast cancer [11]. A higher incidence of TNBC may translate into a higher proportion of the aggressive disease, which may be clinically difficult to target and contributes to higher mortality rates. However, extensive data from India is lacking and there is high variability in the individual studies [5,6].

As there is scant data on TNBC in the rural Indian population, we analyzed our patient data regarding the clinicopathological profiles of and recurrence patterns in patients with TNBCs at our institute where most of the patients are from rural areas.

## Materials and methods

This retrospective study was done at Ganni Subbulakshmi Garu (GSL) Cancer Hospital and Research Centre, Rajahmundry, Andhra Pradesh, India, where most patients come from rural backgrounds. Approval from the GSL Medical College & Hospital Ethics Committee (GSLMC/RC:1199A-EC/1199A-02/2024) was obtained before the study. The case files of all breast cancer patients registered and treated at our center from 2014 to 2019 were retrieved from the medical record department and reviewed. All these patients received treatment at this institute and were consistently managed by a single unit.

The breast cancer diagnosis was primarily based on clinical presentation, imaging (mammogram, ultrasound, or MRI of the breast when needed), and cytopathological studies. Staging was performed using chest X-rays and abdominal ultrasounds for localized disease, with bone scans and computed tomography (CT) or positron emission tomography (PET) scans added for locally advanced and metastatic disease.

Tumors were categorized based on ER, PR, and HER2 status on IHC. At our central pathology laboratory, IHC was done on formalin‑fixed paraffin‑embedded sections using the PathnSitu PolyExcel detection system (Pathnsitu Biotechnologies Pvt. Ltd., Hyderabad, Telangana, India). TNBC is characterized by the absence of expression of the ER and the PR by IHC and human epidermal growth factor receptor overexpression absence either by IHC or absence of amplification by fluorescence in-situ hybridization. Patients with TNBC were identified and their data was retrieved from medical records and analyzed.

Patient characteristics such as age, menopausal status, and family history of cancer were analyzed. Tumor characteristics such as stage, grade, and histology were also studied. Treatment protocols and recurrence rates were evaluated. Staging was conducted following the American Joint Committee on Cancer (AJCC) eighth edition tumor, node, metastasis (TNM) guidelines and the Nottingham histologic score system, which considers tubule formation, nuclear pleomorphism, and mitotic activity, was used for histological grading. Patients were categorized broadly into three groups: early-stage breast cancer (EBC) comprising T1‑2, N0‑1, M0 stages; locally advanced breast cancer (LABC) involving T‑stage ≥T3 and/or N‑stage ≥N2, M0; and metastatic breast cancer (MBC) with evidence of distant metastasis.

Patients were treated according to our institution's protocols based on stage. Chemotherapy was given in a neoadjuvant or adjuvant setting. Commonly used chemotherapy regimens were adriamycin (also called doxorubicin) along with cyclophosphamide (AC) every three weeks for four cycles followed by taxane (T, paclitaxel or docetaxel) every three weeks for four cycles. Surgery was done upfront or after completion of neoadjuvant chemotherapy (NACT) or was sandwiched. Adjuvant radiotherapy was given based on indications.

After the completion of treatment, patients were kept on regular follow-ups as per our institution's protocol. They were followed up every three to four months during the first two years after treatment and every six months thereafter. History was taken and physical examination was done at every follow-up, and imaging with chest X-ray and ultrasound abdomen were done every six months for five years.

Follow-up data of TNBC patients were analyzed to see the pattern and sites of recurrence. Follow-up data till the end of 2022 were analyzed. Surgery to the date of recurrence or last follow-up was defined as disease-free survival (DFS). IBM SPSS Statistics for Windows, Version 20, (Released 2011; IBM Corp., Armonk, New York, United States) was used to plot Kaplan-Meier curves for DFS. Patients with MBC at presentation were excluded from the DFS analysis. Univariate analysis for DFS was done by plotting Kaplan-Meier curves and the log-rank test was used to calculate p-values.

## Results

During the period from 2014 to 2019, a total of 841 patients with breast cancer were enrolled at our institution. Among these cases, 150 were classified as having TNBC, representing 17.8% of the total cases. Detailed patient characteristics can be found in Table 1. The median age at diagnosis was 47 years, ranging from 21 to 72 years. A larger proportion of patients came from rural backgrounds accounting for 143 patients (95.4%), compared to urban residents who were only seven (4.6%). All patients except one were married at the presentation. The predominant presenting symptom observed was a breast lump. The median duration of the lump before diagnosis was three months, with a range of one to 36 months. Upon presentation, 91 patients (60.6%) were identified as postmenopausal, while 59 patients (39.3%) were premenopausal. None of the patients elicited any family history of cancer.

**Table 1:** Patient and tumor characteristics (total number of patients=150)

Characteristic		Number of patients (%)
Age	<50 years	85 (56.6%)
	≥50 years	65 (43.3%)
Menopausal status	Premenopausal	91 (60.6%)
	Postmenopausal	59 (39.3%)
Tumor stage (T)	T1	3 (02%)
	T2	89 (59.3%)
	T3	32 (21.3%)
	T4	26 (17.3%)
Nodal stage (N)	N0	62 (41.3%)
	N1	52 (34.6%)
	N2	22 (14.6%)
	N3	14 (9.3%)
Tumor grade	Grade 1	33 (22%)
	Grade 2	81 (54%)
	Grade 3	36 (24%)
Stage grouping	Stage I	2 (1.3%)
	Stage II	75 (50%)
	Stage III	70 (46.6%)
	Stage IV	3 (2%)

Left-sided tumors were present in 83 patients (55.3%) and were more common than right-sided tumors. The majority of the patients in our study had T2 tumors (59.3%), while T1, T3, and T4 tumors accounted for 2%, 21.3%, and 17.3%, respectively (refer to Table 1 for details). Lymph nodes were involved in 88 patients (58.6%), with N1, N2, and N3 involvement observed in 34.7%, 14.7%, and 9.3% of cases, respectively (Table 1). Among the patients, 77 (51.3%) presented with EBC, 70 (46.6%) with LABC, and only three (2%) had MBC at the time of diagnosis (two had liver metastases and one had brain metastases). Upfront surgery was performed in 119 patients (79.3%), and in the rest of the patients, it was done after NACT. Modified radical mastectomy (MRM) was the most common surgical procedure carried out in 144 patients (96%), while only four (2.6%) underwent breast-conserving surgery (BCS).

Adjuvant chemotherapy was received by 119 patients (79.3%), while 30 (20%) received both NACT and adjuvant chemotherapy (ACT). NACT/ACT regimens were four cycles AC in 130 (86.6%), or four cycles of AC followed by four cycles of paclitaxel in 20 (13.3%) patients. Among those who underwent NACT/ACT, five patients (16.6%) achieved a pathological complete response (pCR). Post-mastectomy radiotherapy was provided to 99 patients (66%). Tumor grading revealed that 33 (22%), 81 (54%), and 36 (24%) patients had Grade 1, Grade 2, and Grade 3 tumors, respectively. Infiltrating ductal carcinoma was the most common histological subtype, occurring in 134 (89.3%) patients. Other common histological subtypes were papillary (seven, 4.6%), mucinous (three, 2%), lobular (three, 2%), and medullary (two, 1.3%).

The median duration of follow-up was 32.8 months (ranging from 12 months to 60 months). A total of 36 patients (24%) experienced recurrence (Table 2). Of these, 10 (30.5%) had EBC at the initial presentation while 26 (72.2%) had LABC. Additionally, three patients experienced a second primary cancer (two patients had ovarian cancer, and one patient developed cancer of the vulva). Most of the patients had a distant failure at multiple sites. The most prevalent site of metastasis was the lungs (14), followed by the bone (seven), the liver (four), and the brain (four). Recurrence rates were notably higher within the first one to three years following the initial diagnosis. The most commonly used second-line chemotherapy regimen was docetaxel.

**Table 2:** Recurrence patterns in TNBC patients TNBC: triple-negative breast cancer

Site of recurrence	Number of patients (%)
Local only	7 (19.4%)
Distant only	24 (66.6%)
Both	2 (5.5%)
Contralateral breast	3 (8.3%)

**Figure 1:**
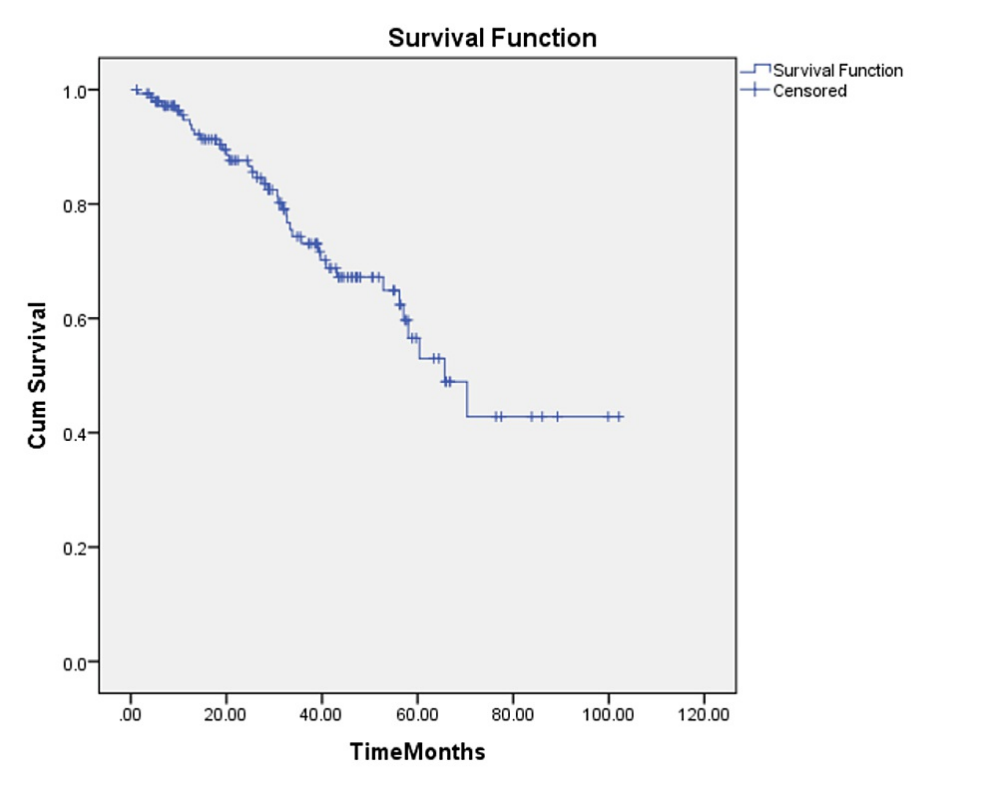
Kaplan-Meier estimate of disease-free survival (DFS) for all patients

The median DFS of TNBC patients was estimated to be 65.6 months (Figure 1).

**Figure 2:**
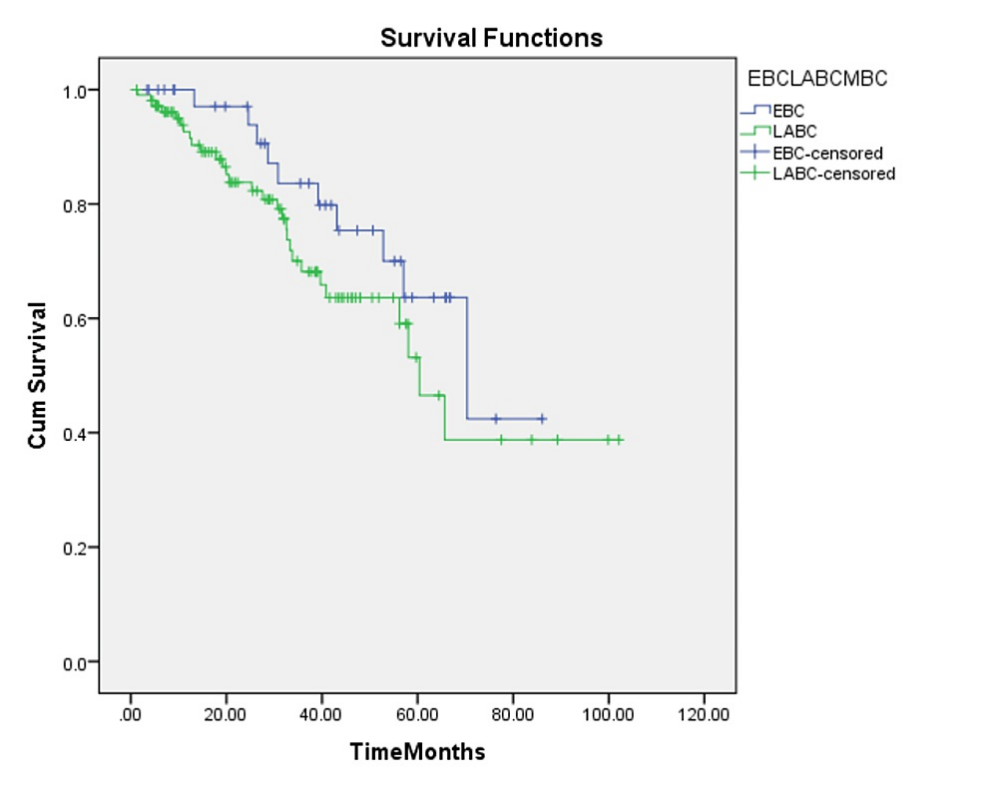
Kaplan-Meier estimated DFS for EBC and LABC EBC: early-stage breast cancer; LABC: locally advanced breast cancer; DFS: disease-free survival

There was no statistically significant difference between the median DFS of EBC and LABC (70.3 months vs. 60.3 months, respectively) (log-rank test, p=0.174) (Figure 2).

## Discussion

TNBC is known for its heterogeneity. While TNBC is often regarded as being more responsive to chemotherapy compared to other types of breast cancer, it also exhibits a more aggressive nature with early recurrence, especially in the first two to three years after diagnosis. For many years, conventional chemotherapy has been the mainstay of the treatment approach for TNBC.

A meta-analysis by Kulkarni et al. showed the combined prevalence of TNBC among patients with breast cancer in India to be around 27% [5]. In our study, TNBCs comprised 17.8% of all breast cancer cases, which aligns with incidence rates reported in studies from Western countries and by other authors in India, ranging from 12% to 27% [2-5]. In similar single institutional studies in India, Lakshmaiah et al., Chintalapani et al., and Suresh et al. reported TNBC prevalence rates of 26%, 19.3%, and 12.5%, respectively [13-15].

The median age at presentation in our study was 47 years. This is comparable to the median age reported in Indian studies. In studies from India by Suresh et al., Lakshmaiah et al., and Chintalapani et al., the median age of the patients was 49, 44.5, and 50 years, respectively [13-15]. The median age of our patients was notably younger, compared to the Western data of 53 years reported by Dent et al. [7]. The discovery of a younger median age likely mirrors the overall trend of breast cancer manifesting at earlier ages in the Indian population compared to Western populations. The majority of our patients were <50 years of age (56.6%) which is reflected by the findings of Lakshmaiah et al where 72.6% of patients were <50 years of age [13]. The majority of our patients were premenopausal (60.6%), which is comparable to the findings of Lakshmaiah et al. (59.5%) and Suresh et al. (48%) [13,15].

Our patients had a median duration of symptoms of less than three months, which is similar to results reported from other Indian TNBC studies [13-15]. The observation that most of the patients in these studies reported a history of a breast lump for less than three months indicates the rapid growth pattern characteristic of TNBCs. The typical risk factors associated with breast cancer were identified in only a small number of patients in this study.

In our study, Stage II diseases were common (50%) followed by Stage III (46.6%). This was similar to the results published by studies by Dent et al. and Prabu et al. [7,16]. Patients with EBC among TNBC patients in our study were 51.3%, while Lakshmaiah et al. and Chintalapani et al. reported findings of 42% and 54%, respectively [13,14]. MBC at presentation in our study was 2%, which is lower than that reported by Lakshmaiah et al. (5.95%) and Chintalapani et al. (5%) [13,14].

Studies have shown that even small tumors in TNBCs carry a significant risk of lymph node positivity [7,17]. This is also shown in our study where the majority of patients were node-positive (58.6%), which is similar to the finding by Chintalapani et al. of 58% [14]. But Lakshmiaih et al. and Suresh et al. have reported that most of the patients were node-negative (36.9% and 65%, respectively) at the presentation in their institutes [13,15].

The majority of patients underwent upfront surgery and only 20% received NACT/ACT. MRM (96%) was the mainstay of surgery in the majority of the patients. The reasons for this varied, including factors such as the extent of disease at the time of diagnosis, concerns about recurrence, and the preferences of either the patient or the surgeon. TNBCs are recognized for their high sensitivity to chemotherapy, resulting in elevated rates of pCR. The observed pCR rate of 16.6% in this study was lower compared to findings reported in studies by Suresh et al. (25%) and Chintalapani et al. (25.8%) [13,15].

Many studies have indicated that patients with TNBC face a higher risk of recurrence. In this study, 36 patients (24%) experienced disease recurrence, primarily manifesting as distant recurrences (in the lung, bone, liver, and brain, in that order), indicating the hematogenous spread characteristic of these cancers. This pattern is similar to that reported by Chintalapani et al. [14]. Recurrence rates were notably higher within the first one to three years following the initial diagnosis. This is in concordance with the observation that the TNBC subgroup has more chance of recurrence within five years [7,10].

The median DFS of TNBC patients in our study was estimated to be 65.6 months. There was no statistically significant difference between the median DFS of EBC and LABC (70.3 months vs. 60.3 months, respectively) (log-rank test, p=0.174). This was in contrast to findings from other studies, which showed a higher DFS for EBC compared to LABC [14,15].

Limitations

There are many limitations to this study as the patients evaluated were treated before the advent of newer recommendations in the management of TNBCs. As per current recommendations, NACT/ACT is the preferable approach in patients with TNBC, which was not the case in our study. None of the patients were tested for BRCA due to resource constraints (up to 20 percent of patients with TNBC may harbor a BRCA mutation).

A further detailed study with a larger patient population treated according to newer recommendations with longer follow-up time is needed to determine if there is any change in the outcomes of TNBC patients at our institute.

## Conclusions

TNBC is known for its heterogeneity. While it is often regarded as being more responsive to chemotherapy compared to other subtypes of breast cancer, TNBCs tend to behave aggressively, basically due to the underlying aggressive tumor biology. Though there are many treatment options for different subtypes of breast cancer, therapeutic modalities are limited for TNBCs. Aggressive tumor biology with limited treatment options denotes a gap in the development of novel strategies to improve outcomes in this subset of TNBC patients.
